# The role of age, theory of mind, and linguistic ability in children’s understanding of ownership

**DOI:** 10.1371/journal.pone.0206591

**Published:** 2018-10-31

**Authors:** Catherine H. McDermott, Nicholaus S. Noles

**Affiliations:** Department of Psychological and Brain Sciences, University of Louisville, Louisville, Kentucky, United States of America; Swinburne University of Technology, AUSTRALIA

## Abstract

The current study replicates and expands prior work on children’s ownership intuitions and explores whether variability in theory of mind and linguistic ability predicts patterns in children’s understanding of ownership. We tested children ages 4 to 6 and found age-related differences in ownership intuitions, but those differences were not significantly predicted by variability in theory of mind or linguistic ability. This report is the first to specifically investigate the cognitive competencies that contribute to the development of mature ownership concepts, and to replicate many of the core findings in the literature.

## Introduction

Concepts of ownership play a central role in human social cognitive development, appearing early in development across cultures [1], and possibly forming the foundation for more mature market behaviors in the future. At an early age, children pay attention to property and they are relatively good at identifying owners [2–5]. However, children exhibit consistent and persistent difficulties when reasoning about property transfers and exchanges [6–8, 2]. Researchers have only recently begun to seriously investigate how ownership concepts develop, revealing a number of interesting developmental patterns [6–7, 9], and cross-cultural differences [10–11]. Below, we will discuss the variability in children’s concepts, and then explore two cognitive competencies that may be related to this variability.

To identify owners, young children often rely on information about an individual’s relationship to property. Specifically, children use proximity, first possession, and control of permission to identify owners [6–7, 12], as well as an object’s history [13, 9, 14]. While children often accurately identify owners, they exhibit a bias to link property rights to an object’s first possessor (i.e., a first possession heuristic), even after a property transfer has occurred [7, 6]. Even when property transfers are acknowledged, children often fail to transfer the relevant property rights along with the property [8].

Prior research indicates that children’s concepts of ownership and property appear early and become more adult-like over the course of early childhood. While some studies have found that children as young as age three are able to accurately identify owners in first-party scenarios [15–16], other studies indicate that children may continue to make errors in identifying owners until age eight [2,7]. Conclusions about exactly when children develop a mature understanding of ownership differ because the relevant studies are often testing different competencies or measuring different behaviors. Studies that highlight early competencies usually employ designs that create strong links between child participants and property assigned to them using well-established cues to ownership, such as proximity, possession, property creation and manipulation, etc. These design choices are appropriate for these studies because they use ownership as a starting condition to test other factors (e.g., the effect of labor on ownership, or children’s tendency to object to property right violations), while studies where children fail to exhibit adult-like behavior present children with third-party scenarios where they must construct the connections between people and property on their own. Thus, the source of developmental differences in ownership understanding are unclear. In addition to differences generated by different methodological approaches, it is possible that children’s understanding of ownership is supported or generated by other cognitive competencies. The purpose of the current study is to explore this third mechanism for development by exploring the relationship between concepts of ownership and two competencies: linguistic ability and theory of mind.

Because so much of our speech is related to identifying owners and labeling property, it is reasonable to suspect that general linguistic ability may be linked to the development of ownership concepts. Indeed, 12-month-olds are provoked by an individual’s use of the pronoun “my” to link a person to their property [17]. Between 18 and 24 months, children’s production of possessive pronouns begins, however the related ownership and property concepts show a much longer developmental trajectory [2]. More generally, this overall improvement in the use of language is associated with improvements in understanding concepts such as desire and need [18]. Indeed, Ross [18] found that very young children’s use of the language relevant to ownership was linked to their use of language about concepts relevant to theory of mind, such as desire and need. These motivational states bring us to the second competency explored in the current study, theory of mind. One problem with studying theory of mind is that it can be difficult to disentangle the influence of other competencies from theory of mind. Thus, if there is a relationship between theory of mind and ownership understanding, having a measure of linguistic ability allows us to disentangle any influences that general linguistic ability may have on theory of mind.

Theory of mind is the understanding of one’s own and others’ mental states [19]. Developing a nuanced theory of mind is an important element of human social cognitive development, as it allows individuals to understand the desires, knowledge, and intentions of others, a task that younger children often have difficulty with [20]. Critically, studies reporting that children find reasoning about property transfers difficult focus on age groups that are actively developing a theory of mind [6–7, 12]. Although it has been proposed that children’s mature understanding of ownership and property transfers develops in conjunction with the development of theory of mind [21–22], little research has directly investigated the relationship between theory of mind and children’s understanding of ownership.

Rochat [21] suggests that ownership understanding develops through six levels, the sixth of which develops around age five in parallel with theory of mind. At this point, children begin to have an understanding of the knowledge and beliefs of others, which allows them to consider what others might be thinking and feeling. He posits that this development of theory of mind is necessary for this final level of ownership understanding, because it is essential to negotiations of value when two parties are transferring or trading property. Incidentally, this final level is also descriptive of situations where children’s generally adult-like intuitions about ownership are the most biased. This narrative is conceptually compelling, but more recent findings reported by Rochat undermine it (see Rochat, et al., 2014 [11]). Rochat and colleagues explored concepts of ownership cross-culturally, but as part of this investigation, they administered a first-order false belief task. Children’s responses to this task did not correlate with their patterns of identifying owners. Rochat and colleagues’ data is suggestive, but they employed only a single real-time false-belief task of the Sally-Anne variety inspired by Wellman, Cross, and Watson [23]. Because this approach offered relatively little power to detect a relationship between ownership and theory of mind, we believe that a broader and more statistically powerful approach is necessary to conclusively link or separate theory of mind and the development of ownership concepts.

Many learning experiences and cognitive competencies may be involved in developing mature ownership concepts. Based on proposed relationships between ownership understanding and other cognitive competencies, the purpose of this paper is to determine whether variability in theory of mind and linguistic ability predicts the degree of development in children’s understanding of ownership, operationalized in the current study as children’s ability to reason about property transfers in an adult-like manner. By characterizing both children’s understanding of theory of mind and their linguistic ability, we can determine more precisely whether the variability in children’s understanding of ownership is related to their developing theory of mind, individual differences in linguistic ability, both competencies, or neither. Because research in the field of ownership is often lacking in theoretical framework, exploring these proposed theories of how ownership concepts may develop could provide a stronger theoretical framework for researchers to reference. In addition, our ownership measures will provide both a replication of previous studies of children’s ownership understanding and a comparison within subjects of the understanding of various concepts of ownership. Previous studies of children’s ownership understanding make different claims about 4- and 5-year-old children’s reasoning about property transfers. In some cases (e.g., when identifying owners in a birthday party scenario) children can reason about property transfers in an adult-like manner and in other cases (e.g., property transfers that do not involve a birthday party) that they cannot [6–7]. In addition, it has been proposed that these adult-like intuitions take longer to develop than previously suggested. Thus, the current study aims to examine these developmental differences in terms of four different property transfers that have previously been investigated.

## Methods

### Participants

Previous studies of both theory of mind and ownership show relatively long trajectories for developing relevant skills. Thus, we tested children ages 4 to 6. This age range encompasses developmental milestones in both the development of ownership concepts and theory of mind. Participants in this study included 54 children (*M*_*age*_ = 5.11, *SD* = .66, females = 28) recruited from homes and schools in urban and suburban areas in and around Louisville, KY, 92% were white, 8% were Asian, and none were Hispanic. Participants were tested individually in a quiet area of their school or in an on-campus testing space.

The University of Louisville Institutional Review Board approved the ethics of this study (IRB Number: 14.0053). Written informed consent was obtained prior to all children’s participation. For participants tested in schools, informed consent documents were sent home and filled out by parents. For children who participated in the on-campus testing space, informed consent was provided by parents when they arrived at the research lab.

### Materials

In order to characterize participant’s understanding of ownership, children were presented with property transfer scenarios based on those used in prior studies, including scenarios depicting serial possession [7], control of permission [12], stealing, and gift-giving [6]. (Example stimuli are available at Databrary.org in a volume sharing the title of this report.) It is important to note that although some studies have found that children exhibit higher rates of first possessor biases when an item starts with the first character [13], the serial possession items in the current study utilized a start-in-middle design [7] to ensure that first possessor biases were indicative of children’s reasoning and not an artifact of our design. The ownership trials were presented to participants on a 15-inch computer screen, in the form of animated scenes depicting each of the corresponding property transfers. Participants were shown 16 trials total, made up of 4 items for each of the 4 different trial types.

For each trial, two characters (of the same gender) were shown, and a property transfer—or lack thereof—was depicted. For example, in the gift-giving trials, children would see two characters on the screen, one on each side. One character started out with a toy and children would be told, “I am going to tell you a story about these two kids. And what’s this? It’s a bear. Well this girl has the bear. And then she gives it to this girl *as a present* [the first possessor moves over to the other character and gives them the toy]. And now this girl has the bear. And now I have a question for you. Whose bear is it?” The movements and positions of characters and items in each vignette were designed to support the narrative of each trial type. At the end of each trial, the characters were positioned on each side of the screen. Children were then asked to identify who owned the property. The characters were cartoon-like figures created using SP-Studio.de [24]. The objects used in these trials were toys, including a ball, airplane, blocks, etc. The final positions of characters were counterbalanced so that the ‘correct’ character (as determined in prior studies) was on each side of the screen in half of the trials. For each trial type, participants saw two items with female characters and two items with male characters, so that the gender of the characters was counterbalanced across the task. The ownership trials were presented in two blocks. One trial of each gender for each trial type was randomly selected in order to populate each block. Presentation order was counterbalanced across participants. The ownership trials were divided in this way in order to hold children’s attention and encourage them to process each item more deeply, as well as to prevent them from falling into a repetitive response pattern. For half of the participants, these trials were presented in the following order: serial possession, gift-giving, control of permission, and stealing. This order was reversed for the remaining participants. Participants’ responses to these scenarios were used to create a composite ownership score reflecting how adult-like children’s intuitions were for each trial type, as well as a more holistic score indicating how adult-like their responses were overall, as compared to data reported in prior studies. Adult-like responses for the explicit property transfers (e.g., gift-giving, control of permission, and stealing) would be those that reflect accurate identification of the owner of after the given property transfer has occurred. For the ambiguous property transfer (e.g., serial possession), the adult like response would be selecting the first-possessor of the item as the owner. As noted in previous studies, when property transfers are ambiguous adults use the first-possessor heuristic and reason that the first person to possess an item is its owner [25].

Participant’s theory of mind was measured using the Theory of Mind Task Battery [26]. The Theory of Mind Task Battery is a 15-item measure that uses vignettes to probe different theory of mind competencies, including emotion recognition, desire-based emotion, standard false belief, etc. Children looked at relevant pictures while the experimenter read the vignettes and recorded children’s responses. For the purpose of this study, four items from two of the vignettes in this scale were excluded because they referenced ownership principles. Participants also completed the Verbal Knowledge and Riddles sections of the Kaufman Brief Intelligence Test [27]. The measure probed children’s linguistic ability and provided a rough estimate of children’s general cognitive ability.

### Procedure

Each child initially completed one block of ownership trials. The pacing of these trials was controlled by the experimenter. Participants then completed the Theory of Mind Task Battery. Next, participants finished the second block of the ownership trials. Lastly, participants completed the KBIT-2.

## Results

### Ownership data

**Fig 1:**
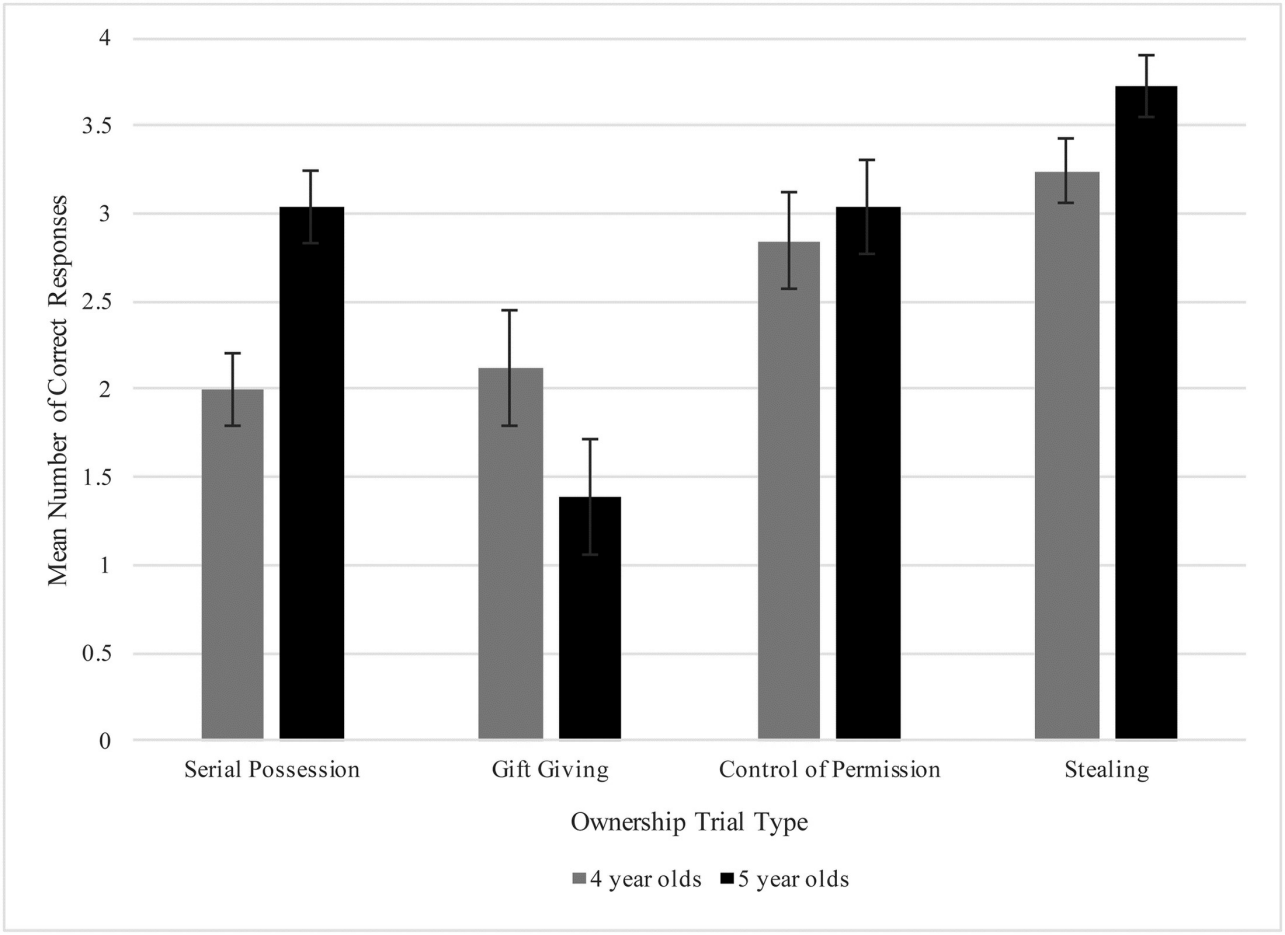
Children’s mean number of adult-like responses by trial-type.

For ownership trials, we created a composite score ranging from 0 to 4 for each trial type, with a 4 being an adult-like response pattern as defined in prior studies (data can be accessed at Databrary.org). A paired samples t-test showed no significant differences in performance between the first and second block of ownership trials, *t*(53) = -.86, *p* = .393. We then conducted a 2 (age: 4- and 5-year-olds) X 4 (trial type: serial possession, gift-giving, control of permission, and stealing) repeated measures ANOVA to examine how participants’ ownership judgments differed by scenario across age groups (Six-year-olds were excluded from these samples because their sample size was not equivalent to the other age groups. Six-year-olds were included in the regression analyses). This analysis revealed a significant main effect of trial type, *F*(3, 147) = 15.00, *p* < .001, $\eta_{p}^{2}=.234$, embedded in a significant interaction between trial type and age, *F*(3, 147) = 3.87, *p* < .05, $\eta_{p}^{2}=.073$. Bonferroni-corrected post-hoc tests revealed that 5-year-old participants responded in a significantly more adult-like manner (*M* = 3.04, *SD* = 1.04) than 4-year-olds on the serial possession trials (*M* = 2.00, *SD* = 1.08), *p* < .01, but differences between age groups for the other three trial types were not significant (Fig 1). Within the 5-year-old age group, participant’s responses were significantly more adult-like on the serial possession (*M* = 3.04, *SD* = 1.04), control of permission (*M* = 3.04, *SD* = 1.43), and stealing trials than on the gift-giving trials (*M* = 1.39, *SD* = 1.70), *p* < .01. Within the 4-year-old age group, participant’s responses were significantly more adult-like on the stealing trials (*M* = 3.24, *SD* = .97) than on the serial possession trials (*M* = 2.00, *SD* = 1.08), *p* < .001.

Within each age group, one-sample t-tests were used to compare the composite means for each trial type to chance responding. In the 4-year old group (*N* = 25, *M*_*age*_ *=* 4.51, *SD* = .29), participants did not differ from chance on the serial possession trials (*M* = 2.00, *SD* = 1.08), *t*(24) = .00, *p* = 1.00, or the gift giving trials (*M* = 2.12, *SD* = 1.64), *t*(24) = .37, *p* = .72, but they responded to control of permission (*M* = 2.84, *SD* = 1.31), *t*(24) = 3.20, *p* < .01, and the stealing trials (*M* = 3.24, *SD* = .97) at rates that were significantly more adult-like than predicted by chance responding, *t*(24) = 6.40, *p* < .01. Five-year-olds’ (*N* = 26, *M*_*age*_ *=* 5.54, *SD* = .28) responses were less adult like than predicted by chance responding on the gift-giving trials (*M* = 1.39, *SD* = 1.70), but this pattern was not significant, *t*(25) = -1.85, *p* = .077. Five-year-olds’ response patterns were significantly above chance on the serial possession (*M* = 3.04, *SD* = 1.04), *t*(25) = 5.10, *p <* .001, control of permission (*M* = 3.04, *SD* = 1.43), *t*(25) = 3.71, *p =* .001, and stealing trials (*M* = 3.73, *SD* = .83), *t*(25) = 10.67, *p <* .001.

### Relationship between cognitive competencies and ownership understanding

There was a positive significant correlation between children’s age and their ownership understanding, *r* = .33, *p* < .01, and between theory of mind scores and linguistic abilities, *r* = .27, *p* < .05, as should be expected, Table 1. Using multiple regression, we tested three models, Table 2 (see Table in S1 Table for summaries of regressions for each type of property transfer). The first model assumed that all three of our variables of interest—age (in years), theory of mind, and linguistic ability—predict children’s performance on ownership trials. This “full” model produced a significant regression equation, *R*^*2*^ = .16, *F*(3, 53) = 3.17, *p* < .05. However, theory of mind scores did not contribute significantly to the model, *p* = .72, nor did linguistic ability, *p* = .10. The second model tested the claim that some combination of linguistic ability and other age-related variance, and not theory of mind, explains the variability in children’s ownership intuitions. This model was also significant, *R*^*2*^ = .16, *F*(2, 53) = 4.77, *p* < .05; however, the contribution of linguistic ability was also not a significant predictor in this model, *p* = .10. The final model that we tested included only age, and was intended to determine, along with the first two models, whether age alone—ostensibly representing variability in experience and/or cognitive ability not measured by our variables of interest—predicts variance in children’s ownership judgments. This model was significant, *R*^*2*^ = .11, *F*(1, 53) = 6.42, *p* < .05. Statistical comparison of these three regression models indicated no significant differences in the predictive power of the models. Since there were no significant differences between the three models and theory of mind and linguistic ability proved to have no significant independent predictive power, we concluded that this final and simplest model was the best fit for these data. Note that all correlations between tested variables were below .5, and the Variance Inflation Factor (VIF) and Tolerance values for these models indicate that multicollinearity was not a problem for interpreting these data.

**Table 1:** Mean theory of mind and linguistic ability scores by age group.

	4-year-olds *M*, *SD*	5-year-olds *M*, *SD*	6-year-olds *M*, *SD*
**Theory of Mind Task Battery**	6.64, 1.78	7.04, 1.22	8.00, 2.65
**KBIT-2 Standardized Score**	113.28, 11.46	108.19, 7.98	111.00, 6.08

**Table 2:** Summary of regression analysis for variables predicting ownership.

Variable	Model 1 *B*, *SEB*, *β*	Model 2 *B*, *SEB*, *β*	Model 3 *B*, *SEB*, *β*
**Exact Age**	1.36, .48, .39*	1.32, .46, .38*	1.16, .46, .33*
**KBIT-2 Standardized Score**	.06, .03, .24	.05, .03, .38	
**Theory of Mind Task Battery**	-.07, .20, -.05		
***R***^***2***^	.16*	.13*	.09*
***F* for change in *R***^***2***^	3.17*	.13	2.89

Table 2 depicts summaries of the three regression models tested.

*p < .05.

## Discussion

The motivation for this study was to examine the relationships between children’s cognitive competencies and their understanding of ownership concepts. Previous research suggested that theory of mind may be playing a role in the development of ownership competencies. Specifically, Rochat [21] suggested that theory of mind is necessary for considering property and ownership in an adult-like manner, but later reversed that position after finding evidence that theory of mind was not related to ownership understanding [11]. The current study uses more sensitive and powerful measures to provide evidence for this second claim, that theory of mind is not a central contributor to the development of mature ownership concepts. Additionally, we demonstrated that variability in children’s linguistic ability similarly played no significant role in explaining the variability in ownership intuitions between children ages 4 to 6. Critically, even when these factors were accounted for, age accounted for a significant independent proportion of the variability between children. Thus, there remain differences between younger and older children that meaningfully bias the way that they think about ownership and property, but that are not predicted by variability in theory of mind or linguistic ability. Specifically, as in prior studies, 4- and 5-year-old children were biased to conserve property with individuals initially identified as the property’s owner.

In addition to our investigation of the relationship between ownership intuitions and cognitive competencies, the current study also created an opportunity to replicate and integrate many findings that form the foundation of the literature on ownership concepts. As in prior studies, 5-year-olds exhibited more adult-like intuitions than 4-year-olds, and their overall response patterns mirror those reported in prior studies with respect to serial possession, control of permission, and stealing [6, 12]. Notably, our results were not perfectly aligned with prior work. While our data was consistent with prior research that found that children do not differ from chance when making ownership judgments in gift-giving scenarios [7], it is inconsistent with others that found that 4- and 5-year-old children performed above chance when making these judgments [6]. These results suggest that children’s intuitions about property transfers (i.e., one person giving an item to another) may, as suggested by Noles and Keil [2], develop more slowly than most expect.

Although the current study provides converging support for the claim that the development of ownership concepts is independent of theory of mind, measures of theory of mind are methodologically entangled with concepts of ownership. Indeed, many measures of theory of mind begin by defining relationships between people and property (e.g., the Sally-Anne task and others, as used by Rochat and colleagues, 2014 [11], and studied in detail by Wellman and Liu, 2004 [28]). Thus, it is possible that some development of theory of mind may actually be attributable to the development of concepts of ownership and property, instead of the of the other way around. This relationship cannot be tested in the current study because items related to ownership were not presented to children. More targeted studies would be required in order to evaluate the contributions of ownership concepts to classic formulations of children’s theory of mind.

In addition to exploring theory of mind, we investigated the contribution of linguistic ability to the development of mature ownership concepts. This effect was not sufficiently consistent or large enough to be considered reliable or qualitatively meaningful, suggesting that mature ownership concepts are relatively independent from linguistic ability. However, it is important that future empirical studies explore the influences that other important domains of cognition (e.g., executive function, nonverbal intelligence, attention, memory, etc.) may have on children’s developing concepts of ownership and property.

## Supporting information

S1 Table Summary of regression analysis for each property transfer. Table depicts summaries of the four individual regression models tested.
(DOCX)

S1 File List of theory of mind skills assessed in theory of mind task battery. File includes a list of the different theory of mind skills assessed by the Theory of Mind Task Battery in the current study.
(DOCX)

S2 File Link to videos of property transfers. File includes a link to videos of the property transfer scenarios shown to children in the current study.
(DOCX)
