# Supplementary material for: The role of age, theory of mind, and linguistic ability in children’s understanding of ownership
Source: PLoS One. 2018 Oct 31;13(10):e0206591. doi: 10.1371/journal.pone.0206591 (PMC6209337; doi:10.1371/journal.pone.0206591)
Supplement: S1 Table — Table depicts summaries of the four individual regression models tested. (DOCX) [file pone.0206591.s001.docx]

**Table 1. Summary of Regression Analysis for each Property Transfer.**

| **Variable** | **Serial Poss.**  ***B SEB β*** | **Gift Giving**  ***B SEB β*** | **Control Perm.**  ***B SEB β*** | **Stealing**  ***B SEB β*** |
| --- | --- | --- | --- | --- |
| **Exact Age** | .84 .24 .47* | -.40 .38 -.15 | .56 .29 .27 | .36 .20 .26 |
| **KBIT-2 Standardized Score** | .01 .02 .07 | .003 .03 .02 | .03 .02 .22 | .01 .01 .14 |
| **Theory of Mind Task Battery** | .01 .10 .02 | -.01 .16 -.01 | .004 .12 .004 | -.08 .09 -.13 |
| ***R^2^*** | .22* | .03 | .10 | .07 |

Table 1 depicts summaries of the four regression models tested. **p* < .05.
