# Supplementary material for: The role of age, theory of mind, and linguistic ability in children’s understanding of ownership
Source: PLoS One. 2018 Oct 31;13(10):e0206591. doi: 10.1371/journal.pone.0206591 (PMC6209337; doi:10.1371/journal.pone.0206591)
Supplement: S1 File — File includes a list of the different theory of mind skills assessed by the Theory of Mind Task Battery in the current study. (DOCX) [file pone.0206591.s002.docx]

**List of Theory of Mind Skills Assessed in Theory of Mind Task Battery**

Emotion Recognition, Desire Based Emotion, Seeing-Leads-to-Knowing, Line of Sight, Inference of Perception-Based Action Task, Standard False Belief, and Message-Desire Discrepant Task. The specific items used in the study are part of a copyrighted measure, which can be obtained at https://www.theoryofmindinventory.com/task-battery/.
