# Supplementary material for: The role of age, theory of mind, and linguistic ability in children’s understanding of ownership
Source: PLoS One. 2018 Oct 31;13(10):e0206591. doi: 10.1371/journal.pone.0206591 (PMC6209337; doi:10.1371/journal.pone.0206591)
Supplement: S2 File — File includes a link to videos of the property transfer scenarios shown to children in the current study. (DOCX) [file pone.0206591.s003.docx]

**Link to Videos of Property Transfers**

<https://louisville.edu/psychology/noles/tom-samples>
